# Reviewed article: Costa AF, Tavares LF, Junior PCPC, Barbosa RBC, Honório OS, Oliveira ACR, Cardoso LO. Adaptation of a pediatric hospital in the city of Niterói, State of Rio de Janeiro, to the recommendations of the Brazilian Dietary Guidelines in 2023. Epidemiol Serv Saude. 2025:34;e20240532

**DOI:** 10.1590/S2237-96222025v34e20240532.b

**Published:** 2025-08-08

**Authors:** Oswaldo Villa-Campos

**Affiliations:** USP, Periodontics

**Table:** 

Rating	Excellent	Good	Average	Below average	Poor
Interest			✓		
Quality			✓		
Originality			✓		
Overall			✓		

**Table:** 

Questionnaire	Yes	No	Not applicable
Does the manuscript contain new and significant information to justify publication?		✓	
Does the Abstract (Summary) clearly and accurately describe the content of the article?		✓	
Is the problem significant and concisely stated?	✓		
Are the methods described comprehensively?		✓	
Are the interpretations and conclusions justified by the results?		✓	
Is adequate reference made to other work in the field?		✓	

## Manuscript Structure

Length of article is: Too long

Number of tables is: Adequate

Number of figures is: Adequate

Please state any conflict(s) of interest that you have in relation to the review of this paper (state “none” if this is not applicable): None

## Comments

Sugestões gerais do manuscrito intitulado: Avaliação e regulamentação do ambiente alimentar: estudo de caso de um hospital pediátrico da rede pública de Niterói, Rio de Janeiro, Brasil.

Similaridades identificadas

1) Verifique similaridades identificadas no relatório iThenticate em anexo e tenha atenção aos trechos com alta similaridade.

2) Verifique na introdução o trecho grifado em verde e mude a escrita para evitar similaridade.

3) Verifique nos resultados: trecho da fase 2 os parágrafos grifados em vermelho presentam alta similaridade com o texto de consulta.

Sugestão itens escrita científica.

1. Revise o título do trabalho para informar o tema principal, delineamento, local e ano(s) da pesquisa

2.Revise o resumo para que tenha linguagem clara e compreensível a todas as pessoas

3. Revise o resumo com chek-list na formatação do texto, revise donde precisa texto em negrito. E elimine texto inecessário

4. Verifique os títulos das ilustrações. Eles devem ser claros, informativos e autossuficientes e com informações as essenciais: local, ano, total de participantes

5. Remova termos pejorativos, generalizações indevidas ou culpabilização dos indivíduos.

6.Revise a fluidez lógica e sequencial das ideias entre parágrafos. As frases precisam ser diretas e sem anunciar o tema do parágrafo. Remova construções como ‘No que se refere a’, ‘Contudo’, ‘Além de’, ‘Neste sentido’ e apresente diretamente o dado.

Resultados

6. Apresentar resultados em sequência que evolua linearmente seguindo a ordem das ilustrações.

7. Evitar texto circular nos resultados. (tratar, mudar de tema e retomar) e excesso de repetição entre texto do resultado e ilustrações.

8. Evitar extrapolações ou interpretações dos resultados que pertencem a discussão.

9.Precisa reorganizar e refazer a escrita dos Resultados conforme as regras da revista.

Discussão

1. Observar o guia de redação pertinente ao delineamento.

2. O primeiro paragrafo da discussão deve resumir os achados, e sem repetir números e nem citar.

3. Apresentar as limitações do estudo, levando em consideraram fontes potenciais de viés no segundo paragrafo

4.interpretar cada resultado relevante e discuti-lo a luz da literatura, esgotar o tema selecionando.

5. Evitar parágrafos sem conexão com a literatura ou que somente a revisam, sem discussão com os resultados

6. o último paragrafo (curto e conciso) deve apresentar a conclusão da pesquisa com base do que pode afirmar a partir dos resultados.

Referencias.

1. Revise a lista de referências verificando se incluir os trabalhos mais relevantes e apoiam as afirmações feitas no texto.

2. Referencias de sites de internet poderiam no ter mesmo peso que um artigo científico.

